# The 2008 Cholera Epidemic in Zimbabwe: Experience of the icddr,b Team in the Field

**DOI:** 10.3329/jhpn.v29i5.8909

**Published:** 2011-10

**Authors:** Sirajuddin Ahmed, Pradip Kumar Bardhan, Anwarul Iqbal, Ramendra Nath Mazumder, Azharul Islam Khan, Md. Sirajul Islam, Abul Kasem Siddique, Alejandro Cravioto

**Affiliations:** icddr,b, GPO Box 128, Dhaka 1000, Bangladesh

**Keywords:** Cholera, Disease outbreaks, Drug therapy, Mortality, Oral rehydration therapy, Zimbabwe

## Abstract

During August 2008–June 2009, an estimated 95,531 suspected cases of cholera and 4,282 deaths due to cholera were reported during the 2008 cholera outbreak in Zimbabwe. Despite the efforts by local and international organizations supported by the Zimbabwean Ministry of Health and Child Welfare in the establishment of cholera treatment centres throughout the country, the case-fatality rate (CFR) was much higher than expected. Over two-thirds of the deaths occurred in areas without access to treatment facilities, with the highest CFRs (>5%) reported from Masvingo, Manicaland, Mashonaland West, Mashonaland East, Midland, and Matabeleland North provinces. Some factors attributing to this high CFR included inappropriate cholera case management with inadequate use of oral rehydration therapy, inappropriate use of antibiotics, and a shortage of experienced healthcare professionals. The breakdown of both potable water and sanitation systems and the widespread contamination of available drinking-water sources were also considered responsible for the rapid and widespread distribution of the epidemic throughout the country. Training of healthcare professionals on appropriate cholera case management and implementation of recommended strategies to reduce the environmental contamination of drinking-water sources could have contributed to the progressive reduction in number of cases and deaths as observed at the end of February 2009.

## INTRODUCTION

In the last 190 years, the world has experienced seven cholera pandemics claiming hundreds of thousands of lives globally (1). The disease still remains a major global challenge to countries where access to safe drinking-water and adequate sanitation could not be assured (2). Over the last few decades, the African continent has become the major focus for endemic and epidemic cholera. The cholera epidemic in Goma, Zaire, in 1994 was one of the worst man-made cholera epidemics in the recent decades (3). Over a decade later, in August 2008, a large cholera epidemic took hold in Zimbabwe. The epidemic started in Chitungwiza district, nearly 25 km southeast of the capital Harare and eventually spread to almost all over the country, affecting 58 of the 62 districts in all 10 provinces by mid-December. During August 2008–mid-January 2009, a country-wide total of 44,272 cholera cases and 2,332 deaths due to cholera were reported to the World Health Organization (WHO) by the surveillance system of the Zimbabwean Ministry of Health and Child Welfare (MoHCW) (4). The overall case-fatality rate (CFR) was 5.3%, and over two-thirds of the deaths were reported from the communities with no access to treatment facilities. Of the 10 provinces, Masvingo, Manicaland, Mashonaland West, Mashonaland East, Midland, and Matabeleland North had the highest CFR of >5% (4). During the same period, 235 cholera treatment centres (CTCs) were established throughout the country by the MoHCW with support from non-governmental organizations (NGOs) and other partners, which provided clinical care to suspected cholera patients (4). With an average of 634 cases and 35 deaths per day (4), there was an urgent need to evaluate the case-management methods practised in the CTCs across the country and to provide appropriate assistance to reduce the number of deaths.

The International Centre for Diarrhoeal Diseases Research, Bangladesh (icddr,b) responded to a request from the WHO's Global Outbreak Alert and Response Network (GOARN) and dispatched a six-member team of cholera experts to Zimbabwe to work with the WHO local office and support the Zimbabwean MoHCW to control the cholera epidemic in the country. The team consisted of three clinicians, one laboratory scientist, and two medical epidemiologists who stayed there from 10 January to 7 February 2009. The objective of the icddr,b team was to assess the cholera situation in Zimbabwe and to provide assistance and reinforce the efforts being undertaken to control the epidemic. This paper describes the observations made by the icddr,b team during this deployment in different CTCs in a number of provinces.

## MATERIALS AND METHODS

Soon after the icddr,b team arrived at Harare, it had discussion with the local WHO staff and delegates from the MoHCW and evaluated the overall cholera situation in the country. Following the discussion, it was decided to make spot-visits to some CTCs located in three different provinces—Harare, Mashonaland West, and Matabeleland North—that had an attack rate of 601, 800, and 597 respectively per 100,000 people since the onset of the epidemic (4). Accordingly, the icddr,b team made spot-visits to 18 CTCs that were providing treatment to patients with acute watery diarrhoea suspected to be cholera in the above three provinces. The assessment included four main activities: (a) detailed discussion with CTC staff members, patients and/or their attendants; (b) evaluation of the physical facilities, including necessary stocks and supplies; (c) review of documents (patient records and case sheets); and (d) observation of day-to-day work of the CTCs and the clinical practices being carried out. A questionnaire was administered to collect information from patients or their attendants regarding the use of oral rehydration salt (ORS) solution or sugar-salt solution (SSS) at home, the distance they had to travel to reach the nearest health facility for treatment, and the source of drinking-water used by the patients. The clinicians of the team assessed the dehydration status of the patients following the WHO criteria.

In addition to assessing and improving the case-management practice, efforts were also made to identify the causative *Vibrio cholerae* strain from samples collected from patients during the visits to the CTCs. No systematic methodology was applied in collecting specimens from patients suffering from acute watery diarrhoea suspected to be cholera. The samples were collected in Cary-Blair transport media and transported to the National Reference Microbiology Laboratory in Harare. *V. cholerae* was identified using standard methods (5). The antibiotic sensitivity pattern was studied using the disc-diffusion method (6,7). Environmental contamination was also evaluated by analyzing water samples collected from five different sources in Harare that were used for drinking and for household purposes. All the water samples were transported in a collection-box with cool packs to the National Microbiology Reference Laboratory in Harare within one hour, and the samples were analyzed using methods described elsewhere (8).

It was realized during the visits to the CTCs that there was an essential need for training of the health professionals in the management and prevention of diarrhoeal diseases, particularly cholera. With assistance from the WHO and the MoHCW, the icddr,b team conducted training sessions for trainers. The trainers included physicians, nurses, paramedics, pharmacists, environmental health officers, and community health professionals. Nine formal day-long training sessions covering all the 10 provinces were held during the last two weeks of the visit. The training courses, comprising lecture sessions, group discussions, and audiovisual presentations, were held to train the groups of health professionals. The topics covered included epidemiology, pathophysiology, diagnosis, clinical management, complications, and prevention of diarrhoeal diseases, concentrating on cholera. Each training session was attended by 15-20 participants selected by the MoHCW.

Computer-based interactive training method was also demonstrated to a core-group of health professionals, comprising physicians and nurses at the Beatrice Road Infectious Diseases Hospital, Harare. The CD (computer disk)-based training programme COTS (cholera outbreak training and shigellosis) was designed to teach the basics of management and treatment of diarrhoeal diseases and many other related topics.

Hands-on-training for effective diarrhoea case management was also provided by the icddr,b team to the health professionals, especially physicians and nurses, working in the CTCs. The nursing staff present during the visits to the CTCs were trained on the correct procedure of obtaining rectal swabs for microbiological culture from diarrhoea patients.

After the departure of the icddr,b team, training programmes were organized at the field level by the trainers who received training so that proper case-management practice was observed uniformly throughout the country.

## RESULTS

### Case-management practices

One of the more consistent observations was the inadequate monitoring of patients following initial rehydration and the continued and overuse of intravenous (IV) fluid. The inadequate use of ORS solution for less severe cases and for those who were already rehydrated by IV fluid was also observed. It was also observed that patients with some dehydration and those already rehydrated with IV fluid were not encouraged to take the ORS solution. Antibiotic administration in accordance with the national cholera-control guidelines was not strictly followed in most CTCs visited.

At the field level, 93 patients were assessed, and their dehydration status was labelled following the WHO criteria (Table 1). Of the patients examined, 95.7% had signs of dehydration, and severe dehydration was noted in 52.7% of the patients. Less than one-third (33.2%) had taken ORS solution or SSS at home before coming to the CTC. More than 60% of the patients had to travel more than 5 km of distance, and 34.4% had to travel a distance of more than 10 km from their home to reach the nearest CTC. The different water sources used by the patients for drinking are also shown in Table 1. More than one-third (37.6%) of the patients interviewed used tap-water for drinking.

**Table 1.** Dehydration status, use of ORS solution at home, distance of CTCs from residence, and source of drinking-water used by patients interviewed by the icddr,b team, 16-23 January 2009

Parameter	Mashonaland West (n=51)		Harare (n=22)		Matabeleland North (n=20)		Total (n=93)	
	No.	%	No.	%	No.	%	No.	%
Dehydration status on admission								
None	2	3.9	2	9.1	0	0	4	4.3
Some	28	56.9	9	40.9	3	15.0	40	43.0
Severe	21	41.2	11	50.0	17	85.0	49	52.7
ORS/SSS taken at home								
Yes	16	31.4	6	27.3	8	40.0	30	32.3
No	35	68.6	16	72.7	12	60.0	63	67.7
Distance (km) of CTCs from home								
<5	19	37.3	9	40.9	6	30.0	34	36.6
5-10	12	21.6	9	40.9	6	30.0	27	29.0
>10	20	39.2	4	18.2	8	40.0	32	34.4
Source of drinking-water								
Tap	17	33.3	16	72.7	2	10.0	35	37.6
Protected well	3	5.9	2	9.1	0	0	5	5.4
Unprotected well	20	39.2	2	9.1	5	25.0	27	29.0
Borehole	4	7.8	2	9.1	2	10.0	8	8.6
River	7	13.7	0	0	11	55.0	18	19.4

CTC=Cholera treatment centre;

ORS=Oral rehydration salt;

SSS=Sugar-salt solution

**Table 2.** Isolation of *Vibrio cholerae* from patient samples

Provinces visited by icddr,b team	No. of specimens collected	*V. cholerae* isolated					
		Ogawa		Inaba		Total	
		No.	%	No.	%	No.	%
Harare	34	12	35.3	4	11.8	16	47.1
Mashonaland West	14	9	64.3	0	0	9	64.3
Matabeleland North	8	2	25.0	3	37.5	5	62.5
Total	56	23	41.1	7	12.5	30	53.6

**Table 3.** Antibiotic resistance pattern of isolated *V. cholerae* strains

Province	No. tested	No. resistant to				
		Tetracycline	Doxycycline	Ciprofloxacin	Erythromycin	Azithromycin
Harare	16	0	0	0	6	0
Mashonaland West	9	2	2	0	7	0
Matabeleland North	5	0	0	0	0	0
Total (%)	30	2 (7)	2 (7)	0	11 (37)	0

### Laboratory support services

Laboratory support was found to be limited, and for many treatment facilities involved in managing the outbreak, this service was not available. Many health personnel were not aware of the local and current antibiotic sensitivity patterns of *V. cholerae.* To identify the causal epidemic strain and to determine the related antimicrobial sensitivity, 56 rectal swabs were collected from patients with suspected cholera, of which 30 (53.6%) samples yielded *V. cholerae* O1 of either Ogawa or Inaba serotype (Table 2). The strains showed variable sensitivity to commonly-used antibiotics, such as tetracycline, doxycycline, and erythromycin. However, they were 100% sensitive to ciprofloxacin and azithromycin (Table 3).

### Environmental contamination

The water sources in Zimbabwe, irrespective of use, appeared to be heavily contaminated. Water samples from various sources in Harare only were collected to detect the contamination profile. All the five water samples indicated widespread faecal contamination, with the drinking-water samples taken from boreholes and dugwells containing high levels of faecal coliforms (26 cfu/100 mL). The tap-water samples were found to be contaminated with total coliforms at a level of 79 cfu/100 mL, with residual chlorine content being 0.06 mg/L, well below the WHO-recommended level of 0.5-0.2 mg/L (9).

## DISCUSSION

In Zimbabwe, cholera is endemic with occasional outbreaks occurring since 1992 (10). Except for 1999 and 2002, the epidemics were managed using available resources without any assistance from the international community (10). Over the years, the disease was kept reasonably under control by implementing intensified prevention strategies. Small outbreaks of cholera that occurred during January-April 2008 in 16 provincial districts did not attract attention because of the inadequacy of disease surveillance and the early warning system due to the apparent deterioration of overall health services in the country (11). However, these small outbreaks returned in August 2008 and spread rapidly all over the country with severe consequences.

It is known that man-made (ethnic and political conflict) and natural disasters are frequently associated with outbreaks of cholera in countries where the disease exists (12-14). The cholera epidemic in Zimbabwe during 2008-2009 was one of the worst in the history of the country and is an example of how man-made disasters can cause degradation in the quality of life due to the destruction of well-established and essential infrastructures.

Most health facilities were found to be operating with fewer numbers of trained health personnel, particularly physicians and nurses, who had left the country due to its worsening economic situation. This resulted in inappropriate and ineffective management of patients, which had a clear direct effect on the treatment outcome during the epidemic. Appropriate rehydration therapy can avert deaths during an epidemic even in a make-shift treatment centre (15). The quick and accurate assessment of the dehydration status of patients, along with a realistic estimation of the volume of fluid needed to be replaced and hydration maintenance, can prevent deaths. Over the past decades, the widespread use of ORS solution at the onset of a diarrhoea episode has contributed significantly to the prevention of cholera-related deaths (3,16). The unavailability of ORS sachets in Zimbabwean communities, along with the unavailability of sugar and clean water, could have contributed to the inadequate and/or non-use of this widely-accepted practice for the management of cholera and other diarrhoeal diseases.

Cholera can cause death within a very short time unless the patient is treated quickly. During the epidemics of cholera, a high death rate is often associated with a delay in seeking treatment at well-managed health facilities (17). The poor communication and transportation system in Zimbabwe, especially in rural areas, prevented many patients from seeking medical care. Environmental contamination of water sources and breakdown of the sanitation system contributed to the spread of cholera throughout the country. Although efforts to identify environmental contamination of water sources in Zimbabwe were limited to Harare only, contamination of water sources by sewage from broken pipes and use of this water by the community in remote areas of the country was consistently observed. A certified laboratory service is essential for the identification of pathogens responsible for epidemics, which provides vital information regarding the pathogen concerned and any related considerations that should be taken when deciding on a suitable antimicrobial therapy. The limited availability of laboratory services in Zimbabwe led to a lack of information for appropriate antibiotic use that possibly contributed to the overall treatment outcome of the disease. Appropriate case management with trained healthcare professionals can greatly contribute to reducing the CFR of cholera (3). The number of cases and deaths was observed to decline at the third week of February 2009 following the departure of the icddr,b team (Fig.). Although the impact of the training programmes was not assessed, it was believed that the training provided during visits to the CTCs could have greatly increased the confidence level and skills of healthcare professionals in managing patients, especially those with severe dehydration.

**Fig.**
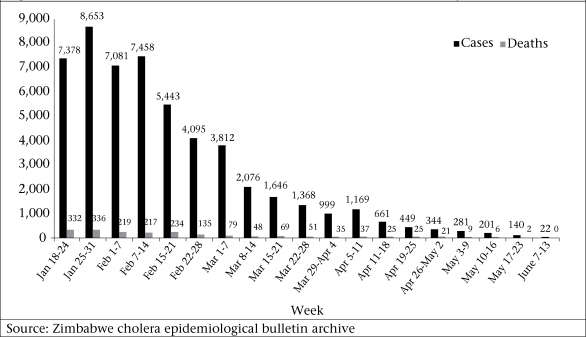
Distribution of cholera cases and related deaths in Zimbabwe, 18 January-13June 2009

### Recommendations

Based on the experience during the cholera epidemic in Zimbabwe, the following recommendations should be put into place in similar situations to manage the spread of the disease and reduce the number of cases and related deaths:

- Uniform standard case management for cholera by all healthcare professionals
- Use of appropriate and effective antimicrobials
- Social mobilization through awareness and the creation of programmes to promote the use of ORS solution by the community at the very onset of disease
- Provision of safe drinking-water and improved sanitation systems
- Development of a certified laboratory service network throughout the country
- Strengthening of the surveillance activities to monitor the disease, and
- Training of essential staff in the management of cholera cases

## ACKNOWLEDGEMENTS

The icddr,b mission to Zimbabwe was supported by the WHO. icddr,b is supported by donors which provide unrestricted support for its operation and research. Current donors providing unrestricted support include: Australian Agency for International Development (AusAID), Government of the People's Republic of Bangladesh, Canadian International Development Agency (CIDA), Swedish International Development Cooperative Agency (Sida), and the Department for International development (DFID), UK. The authors gratefully acknowledge these donors for their support and commitment to icddr,b's research effort.

The icddr,b-Zimbabwe Team expresses its gratitude to the Ministry of Health and Child Welfare, Government of Zimbabwe, and to the staff of the WHO office in Harare for their help.

## References

[1] Barua D, Barua D, Greenough WB (1992). History of cholera.

[2] World Health Organization (2001). Cholera 2000. Wkly Epidemiol Rec.

[3] Siddique AK, Salam A, Islam MS, Akram K, Majumdar RN, Zaman K (1995). Why treatment centres failed to prevent cholera deaths among Rwandan refugees in Goma, Zaire. Lancet.

[4] World Health Organization (2009). Cholera in Zimbabwe. Epidemiol Bull.

[5] World Health Organization (1987). Diarrhoeal Disease Control Programme. Manual for laboratory investigators of acute enteric infections.

[6] Clinical and Laboratory Standards Institute (2009). Performance standards for antimicrobial disk susceptibility tests: approved standard, v. 29. M02-A10, 10th ed..

[7] Bauer AW, Kirby WM, Sherris JC, Truck M (1966). Antibiotic susceptibility testing by a standardized single method. Am J Clin Path.

[8] Sirajul Islam M, Brooks AM, Kabir MS, Jahid IK, Shafiqul Islam M, Goswami D (2007). Faecal contamination of drinking water sources of Dhaka city during 2004 flood in Bangladesh and use of disinfectants for water treatment. J Appl Microbiol.

[9] World Health Organization (2010). Disinfection: session objectives. Guidelines for drinking-water quality: training materials.

[10] World Health Organization (2008). Cholera in Zimbabwe. Wkly Epidemiol Rec.

[11] World Health Organization (2008). Cholera in Zimbabwe:. Epidemiol Bull.

[12] Kirgia JM, Sambo LG, Yokouide A, Soumbey-Alley E, Muthuri LK, Kirgia DG (2009). Economic burden of cholera in the WHO African region. BMC Int Health Hum Rights.

[13] Siddique AK, Islam Q, Akram K, Mazumder Y, Mitra A, Eusof A (1989). Cholera Epidemic and natural disasters: where is the link?. Trop Geog Med.

[14] Watson JT, Gayer M, Conolly MA (2007). Epidemics after natural disasters. Emerg Infect Dis.

[15] Siddique AK, Mutsuddy P, Islam Q, Majumder Y, Akram K, Zaman K (1990). Makeshift treatment centre during a cholera epidemic in Bangladesh. Trop Doct.

[16] Siddique AK, Zaman K, Baqui AH, Akram K, Mutsuddy P, Eusof A (1992). Cholera epidemics in Bangladesh: 1985–1991.. J Diarrhoeal Dis Res.

[17] Goma Epidemiology Group (1995). Public health impact of Rwandan refugee crisis: what happened in Goma, Zaire in July 1994?. Lancet.

